# Quantifying entanglement in a 68-billion-dimensional quantum state space

**DOI:** 10.1038/s41467-019-10810-z

**Published:** 2019-06-25

**Authors:** James Schneeloch, Christopher C. Tison, Michael L. Fanto, Paul M. Alsing, Gregory A. Howland

**Affiliations:** 1 Air Force Research Laboratory, Information Directorate, Rome, NY 13441 USA; 2 Department of Physics, Florida Atlantic University, Boca Raton, FL 33431 USA; 3 Quanterion Solutions Incorporated, Utica, NY 13502 USA; 4 Rochester Institute of Technology, Rochester, NY 14623 USA

**Keywords:** Quantum optics, Quantum information, Single photons and quantum effects

## Abstract

Entanglement is the powerful and enigmatic resource central to quantum information processing, which promises capabilities in computing, simulation, secure communication, and metrology beyond what is possible for classical devices. Exactly quantifying the entanglement of an unknown system requires completely determining its quantum state, a task which demands an intractable number of measurements even for modestly-sized systems. Here we demonstrate a method for rigorously quantifying high-dimensional entanglement from extremely limited data. We improve an entropic, quantitative entanglement witness to operate directly on compressed experimental data acquired via an adaptive, multilevel sampling procedure. Only 6,456 measurements are needed to certify an entanglement-of-formation of 7.11 ± .04 ebits shared by two spatially-entangled photons. With a Hilbert space exceeding 68 billion dimensions, we need 20-million-times fewer measurements than the uncompressed approach and 10^18^-times fewer measurements than tomography. Our technique offers a universal method for quantifying entanglement in any large quantum system shared by two parties.

## Abstract (web summary)

Quantifying entanglement in a large quantum system requires an intractable number of measurements. Here, the authors demonstrate a method for certifying entanglement from extremely undersampled data by combining a new quantitative entanglement witness with an adaptive sampling procedure.

## Introduction

Achieving a quantum advantage for information processing requires scaling quantum systems to sizes that can provide significant quantum resources, including entanglement. Large quantum systems are now realized across many platforms, including atomic simulators beyond 50 qubits^1–3^, nascent superconducting and trapped-ion-based quantum computers^4,5^, integrated-photonic circuits^6–10^, and photon pairs entangled in high-dimensional variables^11–16^.

As quantum-information-based technologies mature, it will become useful to separate the physical layer providing quantum resources (e.g., trapped ions, photons) from the logical layer that utilizes those resources. For example, many imperfect qubits may form one logical qubit^17,18^ or thousands of atoms may coherently act as a single-photon quantum memory^19,20^. As with classical communication and computing, protocols and algorithms will be implemented in the logical layer with minimal concern for the underlying platform. Because real-world systems are varied and imperfect, the quantum resources they provide must be characterized before use^17^.

Certifying an amount of entanglement in a large quantum system is an essential but daunting task. While entanglement witnesses^21,22^ and Bell tests^23^ can reveal entanglement’s presence, quantification generally requires a full estimation of the quantum state^24^. Beyond moderately sized states, the number of parameters to physically measure (i.e., the number of the measurements) becomes overwhelming, making this approach unviable for current and future large-scale quantum technologies.

Any practical method for quantitative entanglement certification must require only limited data. Two ideas can dramatically reduce the needed measurement resources. First is the development of quantitative entanglement witnesses, which bound the amount of entanglement without full state estimation^25–28^. In a recent landmark experiment, 4.1 entangled bits (ebits) of high-dimensional biphoton entanglement was certified using partial state estimation^29^. One ebit describes the amount of entanglement in a maximally entangled, two-qubit state^24^.

Second, prior knowledge can be exploited to economize sampling. Certain features, or structure, are expected in specific systems. In highly entangled quantum systems, for example, some observables should be highly correlated, the density matrix will be low rank, or the state may be nearly pure. Such assumptions can be paired with numerical optimization to recover signals sampled below the Nyquist limit. One popular technique is compressed sensing^30^, which has massively disrupted conventional thinking about sampling. Applied to quantum systems, compressed sensing reduced measurement resources significantly for tasks, including tomography^31–37^ and witnessing entanglement^38,39^.

Computational recovery techniques have substantial downsides. Because they are estimation techniques, conclusions drawn from their results are contingent on the veracity of the initial assumptions. They are therefore unsuitable for closing loopholes or verifying security. Numerical solvers are often proven correct under limited noise models and require hand-tuned parameters, potentially adding artifacts and complicating error analysis. Finally, the computational resources needed become prohibitive in very large systems. The largest quantum systems characterized using these approaches remain considerably smaller than state-of-the-art.

Here we provide an approach to entanglement quantification that overcomes these downsides. First, we improve an entropic, quantitative entanglement witness to operate on arbitrarily downsampled data. Then we develop an adaptive, multilevel sampling procedure to rapidly obtain compressed distributions suitable for the witness. Crucially, our sampling assumptions are independent of the entanglement certification, so our method can guarantee security. Because we avoid numerical optimization, error analysis is straightforward and few computational resources are needed.

## Results

### Entropic witnesses of high-dimensional entanglement

Entanglement is revealed when subsystems of a quantum state are specially correlated. A common situation divides a system between two parties, Alice and Bob, who make local measurements on their portion. Given two mutually unbiased, continuous observables $\widehat {\mathbf{x}}$ and $\widehat {\mathbf{k}}$, they can measure discrete joint probability distributions *P*(**X**_a_, **X**_b_) and *P*(**K**_a_, **K**_b_) by discretizing to pixel sizes Δ_*X*_ and Δ_*K*_. Here bold notation indicates that **X** and **K** may (though need not) represent multidimensional coordinates. For example, **X** and **K** might represent cartesian position and momentum that can be decomposed into horizontal and vertical components such that **X** = (*X*, *Y*) and **K** = (*K*^(*x*)^, *K*^(*y*)^).

A recent, quantitative entanglement witness^40^ uses these distributions to certify an amount of entanglement:

1 $$d\log _2\left( {\frac{{2\pi }}{{\Delta _{\mathrm{X}}\Delta _{\mathrm{K}}}}} \right) - H({\mathbf{X}}_{\mathrm{a}}|{\mathbf{X}}_{\mathrm{b}}) - H({\mathbf{K}}_{\mathrm{a}}|{\mathbf{K}}_{\mathrm{b}}) \le E_{\mathrm{f}},$$

where, for example, *H*(**A**|**B**) is the conditional Shannon entropy for *P*(**A**, **B**). *E*_f_ is the entanglement of formation, a measure describing the average number of Bell pairs required to synthesize the state. Equation (1) does not require full-state estimation but depends on an informed choice of $\widehat {\mathbf{x}}$ and $\widehat {\mathbf{k}}$. Still, in large systems, measuring these joint distributions remains oppressive. For example, if **X**_a_ has 100 possible outcomes, determining *P*(**X**_a_, **X**_b_) takes 100^2^ joint measurements. Describing quantum uncertainty with information-theoretic quantities is increasingly popular^41,42^. Entropies naturally link physical and logical layers and have useful mathematical properties. In particular, many approximations to the joint distributions can only increase conditional entropy. Because Eq. (1) bounds *E*_f_ from below, any such substitution is valid.

### Improving an entropic entanglement witnesses for use with limited data

We use two entropic shortcuts to improve the entanglement witness. First, if the system is highly entangled, and $\widehat {\mathbf{x}}$ and $\widehat {\mathbf{k}}$ are well chosen, the joint distributions will be highly correlated; a measurement outcome for **X**_a_ should correlate to few outcomes for **X**_b_. The distributions are therefore highly compressible. Consider replacing arbitrary groups of elements in *P*(**X**_a_, **X**_b_) with their average values to form a multilevel, compressed estimate $\tilde P({\mathbf{X}}_{\mathrm{a}},{\mathbf{X}}_{\mathrm{b}})$. By multilevel, we mean that the new, estimated distribution will appear as if it was sampled with varying resolution—fine detail in some regions and coarse detail in others. Because coarse graining cannot decrease conditional entropy, Eq. (1) remains valid for $\tilde P({\mathbf{X}}_{\mathrm{a}},{\mathbf{X}}_{\mathrm{b}})$ and $\tilde P({\mathbf{K}}_{\mathrm{a}},{\mathbf{K}}_{\mathrm{b}})$ (see Supplemental Material: Proof arbitrary coarse-graining cannot decrease conditional entropy).

Good estimates for $\tilde P({\mathbf{X}}_{\mathrm{a}},{\mathbf{X}}_{\mathrm{b}})$ and $\tilde P({\mathbf{K}}_{\mathrm{a}},{\mathbf{K}}_{\mathrm{b}})$ can be efficiently measured by sampling at high resolution in correlated regions and low resolution elsewhere. Note that the original (*P*) and estimate ($\tilde P$)) are full correlation matrices with *N* elements, but only $M \ll N$ values measured to specify $\tilde P$. The witness is valid for arbitrary downsampling; it works best when the approximate and actual distributions are most similar but can never overestimate *E*_f_ or allow false positives.

Second, if the observables are multi-dimensional such that they can be decomposed into *d* marginal, component observables (e.g., horizontal and vertical components) $\widehat {\mathbf{x}} = (\hat x^{(1)},\hat x^{(2)},...,\hat x^{(d)})$ (similar for $\widehat {\mathbf{k}}$), the conditional entropies have the property

2 $$H({\mathbf{X}}_{\mathrm{a}}|{\mathbf{X}}_{\mathrm{b}}) \le \mathop {\sum}\limits_{i=1}^d H (X_{\mathrm{a}}^{(i)}|X_{\mathrm{b}}^{(i)}),$$

with equality when *P*(**X**_a_, **X**_b_) is separable. If we expect nearly separable joint-distributions, the reduced, marginal joint-distributions $P(X_{\mathrm{a}}^{(i)},X_{\mathrm{b}}^{(i)})$ can be separately measured but still capture nearly all of the correlations present. For example, in a two-dimensional cartesian scenario, we might separately measure horizontal correlations *P*(*X*_a_, *X*_b_), $P(K_{\mathrm{a}}^{(x)},K_{\mathrm{b}}^{(x)})$ and vertical correlations *P*(*Y*_a_, *Y*_b_), $P(K_{\mathrm{a}}^{(y)},K_{\mathrm{b}}^{(y)})$. For *d*-component observables, this is a *d*th power reduction in the number of measurements. Like the first shortcut, this approximation also cannot overestimate *E*_f_.

Combining both improvements, our new quantitative entanglement witness is

3 $$\mathop {\sum}\limits_{i = 1}^d {\left[ {\log _2\left( {\frac{{2\pi }}{{\Delta _{\mathrm{X}}^{(i)}\Delta _{\mathrm{K}}^{(i)}}}} \right) - \tilde H(X_{\mathrm{a}}^{(i)}|X_{\mathrm{b}}^{(i)}) - \tilde H(K_{\mathrm{a}}^{(i)}|K_{\mathrm{b}}^{(i)})} \right] \le E_{\mathrm{f}}.}$$

### Proof-of-concept experimental set-up

**Fig. 1:**
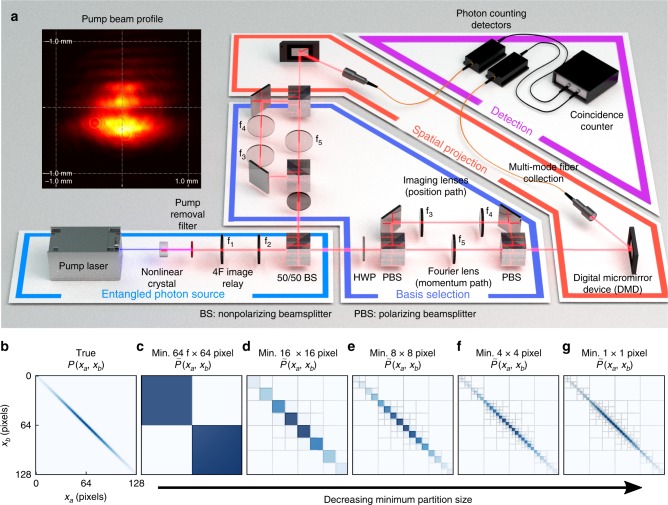
Experimental set-up for adaptive measurements. **a** An entangled photon source produces spatially entangled photon pairs, which are separated and routed through basis selection optics that switch between measuring transverse-position or transverse-momentum. Computer-controlled digital micromirror devices and photon-counting detectors perform joint spatial projections at up to 512 × 512 pixel resolution. **b** shows a simulated, true position joint-distribution of *P*(*X*_a_, *X*_b_) at 128 × 128 pixel resolution, while **c**–**g** show its simulated, adaptively decomposed estimate $\tilde P(X_{\mathrm{a}},X_{\mathrm{b}})$ as it is refined to higher detail via quad-tree decomposition. When the joint-intensity in a block exceeds a user-defined threshold, it is split into four sub-quadrants and the process is recursively repeated, rapidly partitioning the space to obtain a compressed distribution from very few measurements

As a test experimental system, we use photon pairs entangled in their transverse-spatial degrees of freedom^43,44^, where the transverse plane is perpendicular to the optic axis. Our test bed, given in Fig. 1a, creates photon pairs via spontaneous parametric downconversion (see “Methods”). Generated photons are positively correlated in transverse-position and anti-correlated in transverse-momentum. This state closely approximates the original form of the Einstein–Podolsky–Rosen paradox. Because position $\widehat {\mathbf{x}} = (\hat x,\hat y)$ and momentum $\widehat {\mathbf{k}} = (\hat k^{(x)},\hat k^{(y)})$ (where $\widehat {\mathbf{k}} = \widehat {\mathbf{p}}/\hbar$) observables are continuous, this state is very high dimensional.

After creation, the twin photons are separated at a beam splitter and enter identical measurement apparatuses, where a basis selection system allows for interrogating position or momentum. A digital micromirror device (DMD)—an array of individually addressable micromirrors—is placed in the output plane. By placing patterns on the signal and idler DMDs and using coincidence detection, rectangular regions of the position or momentum joint-distributions are sampled at arbitrary resolution.

### Adaptive, multi-level data acquisition

We measure joint-distributions $\tilde P(X_{\mathrm{a}},X_{\mathrm{b}})$, $\tilde P(Y_{\mathrm{a}},Y_{\mathrm{b}})$, $\tilde P(K_{\mathrm{a}}^{(x)},K_{\mathrm{b}}^{(x)})$, and $\tilde P(K_a^{(y)},K_{\mathrm{b}}^{(y)})$. Finding compressed distributions requires a multilevel partitioning of the joint-space that is not known a priori. Our adaptive approach is inspired by quad-tree image compression^45^. An example is shown in Fig. 1b–g. First, all DMD mirrors are directed toward the detector to obtain a total coincidence rate *R*_T_. Then the joint-space is divided into four quadrants (c), which are independently sampled. If the count rate in the *i*th quadrant exceeds a threshold *αR*_T_ (0 ≤ *α* ≤ 1), the region is recursively split and the process is repeated. The algorithm rapidly identifies important regions of the joint-space for high-resolution sampling.

**Fig. 2:**
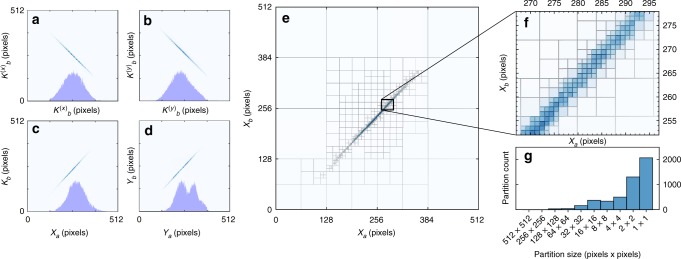
Measured joint probability distributions at 512 × 512 pixel resolution. **a**–**d** show the four estimated joint probability distributions with their single-party marginal distributions overlaid, showing tight correlations. **e** shows an enlarged version of $\tilde P(X_{\mathrm{a}},X_{\mathrm{b}})$ overlaid with the adaptive partitioning, with **f** showing a small central region to see fine detail. The histogram **g** shows the number of partitions as a function of their area. Only 6456 measurements are needed instead of 2 × 512^4^

We set the maximum resolution of our system to 512 × 512 pixels-per-photon for a 512^4^-dimensional joint-space. The recovered joint-distributions in position and momentum are given in Fig. 2a–d. Figure 2e, f show $\tilde P(X_{\mathrm{a}},X_{\mathrm{b}})$ with the partitioning overlaid. These display the expected strong position and momentum correlations. A histogram showing the number of partitions at various scales is given in Fig. 2g; most partitions are either 1 × 1 or 2 × 2 pixels in size. Only 6456 partitions are needed to accurately cover the 512^4^-dimensional space—an astonishing 20-million-fold improvement versus using the unimproved witness. Over 10^21^ measurements are needed to perform full, unbiased tomography.

**Fig. 3:**
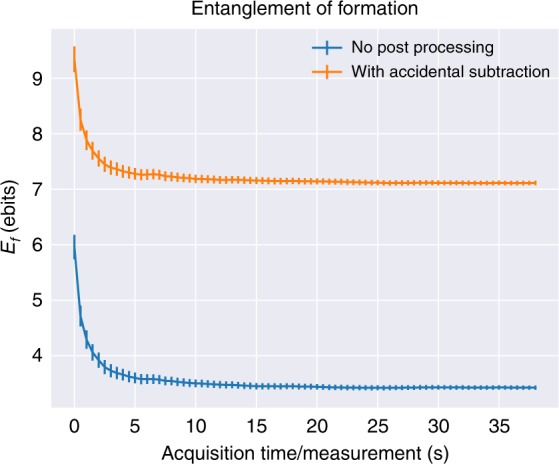
Entanglement quantification versus acquisition time. The entanglement of formation *E*_f_ is given as a function of acquisition time-per-partition for unaltered coincidence data and accidental-subtracted data. Error bars enclosing two standard deviations are determined by propagation of error from photon-counting statistics. We confirm the validity of this error analysis strategy via Monte Carlo simulation in Supplemental Material: Monte Carlo error analysis (see Supplemental Fig. 1)

The entanglement witness (Eq. (3)) applied to the data in Fig. 2 is shown in Fig. 3. For short acquisition times, there is a systematic bias toward estimating a large *E*_f_. This occurs because many of the poorly correlated regions have not yet accumulated any detection events, resulting in a systematic bias toward low conditional entropies. Statistical error is low in this region because the highly correlated regions have high count rates and rapidly reach statistical significance. With additional measurement time, the initial bias diminishes and statistical error decreases. To our knowledge, 7.11 ± .04 ebits is the largest quantity of entanglement experimentally certified in a quantum system. More than 14 maximally pairwise-entangled logical qubits are needed to describe an equal amount of entanglement. We do not require advanced post-processing such as numerical optimization, estimation, or noise reduction; however, we do post-select on coincident detection events and optionally subtract accidental coincidences (see “Methods”). Our witness does not explicitly require any post-processing and is suitable for use in adversarial scenarios given a pristine experimental system.

**Fig. 4:**
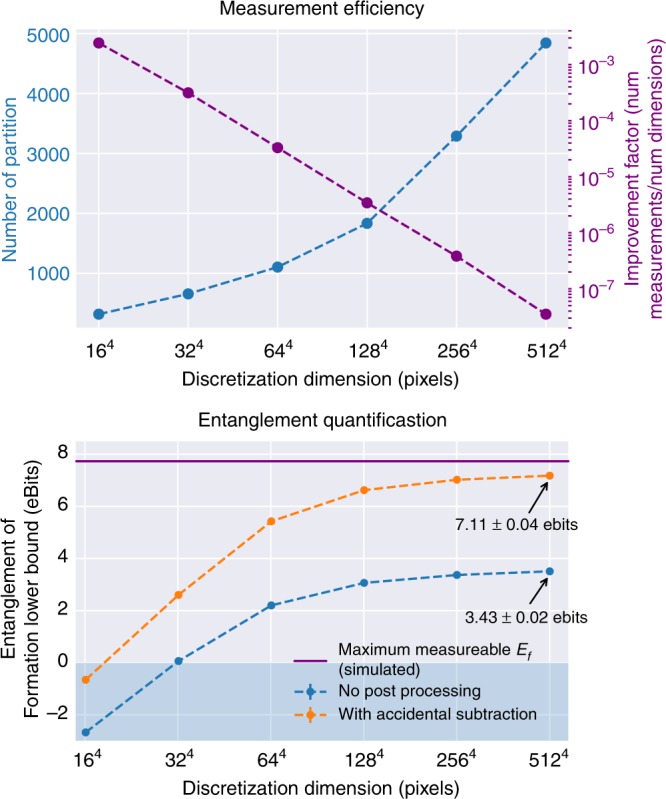
Entanglement quantification versus maximum resolution. **a** shows the number of partitions required as a function of maximum allowed resolution and the improvement over the uncompressed approach. **b** shows the amount of entanglement captured as the maximum resolution increases. We see the progressive nature of the technique, which witnesses entanglement with few measurements at low resolution but more accurately quantifies it with further refinement. Our results approach the ideal maximum measurable value *E*_f_ = 7.68 ebits for our source

The performance of our technique as a function of maximum discretization resolution is shown in Fig. 4. Figure 4a shows the approximate distribution partition number as a function of discretization dimension and the improvement factor over naive sampling. Figure 4b shows the certified *E*_f_, with and without accidental subtraction, along with the ideal *E*_f_ for our source under a double-Gaussian approximation^44^. Because our pump laser is not Gaussian (Fig. 1a), the actual *E*_f_ is slightly less but difficult to simulate. Error bars enclosing two standard deviations are scarcely visible. For low resolution, <1000 measurements witness entanglement. Progressively refining to higher effective resolution allows more entanglement to be certified until the maximum is reached.

## Discussion

We have shown an efficient method for performing information-based entanglement certification in a very large quantum system. An alternative, important metric for quantifying entanglement in high-dimensional systems is the entanglement dimensionality, or Schmidt rank, which describes the number of modes over which the entanglement is distributed^22,46–48^. In contrast, entanglement measures quantify entanglement as a resource of entangled bits without regard for their distribution. Efficiently certifying the entanglement dimensionality faces many of the same problems as certifying a number ebits, such as the intractability of full tomography and the desire to avoid side effects from prior assumptions. Recently, Bavaresco et al. used measurements in only two bases to efficiently certify over nine entangled dimensions between orbital-angular-momentum entangled photon pairs without special assumptions about the underlying state^49^.

The number of entangled dimensions and the number of entangled bits are complementary but distinct characterizations of entanglement^50^. If a density matrix cannot be decomposed into pure states with Schmidt rank <*d*, then the state is at least *d*-dimensionally entangled. However, a *d*-dimensional entangled state may possess an arbitrarily small amount of entanglement. Consider a system with a large Schmidt rank but where one coefficient of the Schmidt decomposition is much larger than the others. This system will have a large entanglement dimensionality but require few entangled bits to synthesize. In this way, a given entanglement dimensionality *D* provides an upper bound on the entanglement of formation *E*_f_ such that $0 < E_{\mathrm{f}} \le \log _2D$. In contrast, a given *E*_f_ provides a lower bound to the entanglement dimensionality $D \ge 2^{E_{\mathrm{f}}}$, describing the situation where all *D* dimensions are maximally entangled. Our quantitative witness therefore also certifies entanglement dimensionality but may dramatically underestimate when the target system is not near-maximally entangled (e.g., with additive noise or non-uniform marginals). In our case, we certify 2^7.11^ ≥ 138 maximally entangled dimensions with background subtraction and 2^3.43^ ≥ 10 maximally entangled dimensions without background subtraction. To our knowledge, 10 entangled dimensions is the largest certified entanglement dimensionality without assumptions about the state.

Our approach shows a path forward for certifying quantum resources in large quantum systems, where we exploit prior knowledge without conventional downsides. We show the power of an information-theoretic approach to characterizing quantum systems, and how compression can be leveraged without computational signal recovery. Though the method presented here is limited to Einstein–Podolsky–Rosen-type systems where entanglement is shared by two parties, we expect that similar techniques for many-body systems utilizing higher-order correlations will soon follow.

## Methods

### Experimental apparatus

The 810-nm, spatially entangled photon pairs are produced via spontaneous parametric downconversion (SPDC)^44^. The pump laser is a 405-nm diode laser (CrystaLaser DL405-025-SO) attenuated to 7.9 mW with a 356 μm (*x*) × 334 μm (*y*) beam waist. A spectral clean-up filter (Semrock Versachrome TBP01-400/16) removes unwanted the 810-nm light. The pump laser is not spatially filtered. The nonlinear crystal is a 3-mm-long BiBO crystal oriented for type-I, degenerate, collinear SPDC. The crystal is held at 32.3 °C in an oven for long-term stability. A low-pass interference filter (Semrock LP442) removes remaining pump light, followed by a telescope relay system (*f*_1_ = 50 mm, *f*_2_ = 100 mm) that magnifies the SPDC field ≈2×. A half-waveplate and polarizing beamsplitter allow switching between imaging ($\widehat {\mathbf{x}}$) and Fourier-transforming ($\widehat {\mathbf{k}}$) beam-paths; a beam block is placed in the unused path.

The DMDs (TI Lightcrafter 4500) are computer controlled via a digital video port (HDMI). A 512 × 1024 physical-pixel area was used for data given in this manuscript. Because the DMD has twice the vertical pixel density, this corresponds to a square area. The 10-mm effective focal length, aspheric lenses (Thorlabs AC080-010) couple light into 100 micron core multi-mode fibers connected to photon-counting detector modules (Excelitas SPCM-AQ4C-10). The 810/10 nm bandpass filters (Thorlabs FBS810-10) are placed before the fiber coupling. A time-correlated single-photon counting module (PicoQuant HydraHarp400) produces histograms of photon-pair relative arrival times. We post-select on coincident detections within a 1-ns coincidence window centered on the histogram peak. With all DMD mirrors pointed toward the detectors, there are approximately 26,400 total coincidences/s.

### Data collection

The apparatus must be adjusted to separately measure the four reduced, joint-probability distributions *P*(*X*_a_, *X*_b_), *P*(*Y*_a_, *Y*_b_), $P(K_{\mathrm{a}}^{(x)},K_{\mathrm{b}}^{(x)})$, and $P(K_{\mathrm{a}}^{(y)},K_{\mathrm{b}}^{(y)})$. For example, to access the horizontal, joint-position distribution *P*(*X*_a_, *X*_b_), we adjust the half-waveplates to direct light down the imaging beam-paths so the DMDs lie in an image plane of the nonlinear crystal. To access a particular, rectangular element of the distribution, local, one-dimensional “top-hat” patterns are placed on signal (a) and idler (b) DMDs that only vary horizontally. In the regions where light should be directed to the detectors, all vertical pixels are used. The local images’ outer-product defines the rectangular region of the joint-space *P*(*X*_a_, *X*_b_) that is being sampled.

To instead access the vertical, joint-position distribution *P*(*Y*_a_, *Y*_b_), local DMD patterns are used that only vary vertically. The joint-momentum distributions are similarly sampled, with the half-waveplates instead adjusted to send light down the Fourier-transforming optical path so that the DMDs sit in the far-field of the nonlinear crystal.

### Adaptive sampling algorithm

For each configuration, experimental data are stored in nodes in a quad-tree decomposition of *P* whose levels describe increasingly fine detail. The *i*th node corresponds to a square area of $\tilde P$ at location $(x_{\mathrm{a}}^i,x_{\mathrm{b}}^i)$ with span $w_{\mathrm{a}}^i = w_{\mathrm{b}}^i = w$. Nodes are sampled by placing the corresponding, one-dimensional local patterns on the DMDs and generating a coincidence histogram during acquisition time *T*_a_ = 0.5 s. Coincidences *C*_*i*_ are counted within a 1-ns coincidence window centered on the coincidence peak; accidental coincidences *A*_*i*_ are counted in a 1-ns window displaced 2 ns from the coincidence window. Coincidence and accidental values are appended to a list each time the node is sampled. The estimated count rate $R_i = \left\langle {C_i} \right\rangle /\epsilon _iT_{\mathrm{a}}$, where $\epsilon _i$ is a calibrated, relative fiber coupling efficiency. Optionally, *A*_*i*_ can be subtracted from *C*_*i*_ for accidental removal. Uncertainty is computed by assuming Poissonian counting statistics for *C*_*i*_ and *A*_*i*_ and applying standard, algebraic propagation of error through the calculation of the entanglement quantity (Eq. (3)).

The data collection algorithm consists of a partitioning phase followed by an iterative phase. During partitioning, the algorithm repeatedly iterates through a scan-list of leaves of the tree. Node *i* is considered stable when sgn(*αR*_T_ − *R*_*i*_) is known to at least *β* standard deviations of certainty, where splitting threshold *α* (0 ≤ *α* ≤ 1) and stability criterion *β* are user-chosen heuristics. Stable nodes are no longer measured. If a node is stable and *R*_*i*_ ≥ *αR*_T_, the node is split into four equal-sized sub-quadrants, which are initially unstable and added to the scan-list. Optionally, a maximum resolution (maximum tree depth) may be set.

The transition to the iterative phase occurs when the percentage of unstable leaves is <*Γ*, a user-chosen parameter. At this point, stability is ignored and all leaf nodes are scanned repeatedly and guaranteed to have the same total acquisition time. Various final stopping criteria can be used; we chose a fixed total run time. Note that heuristic parameters *α*, *β*, and *γ* may be changed during operation if desired. For the data shown in this manuscript, *α* = 0.002, *β* = 2, and *Γ* = 0.15 with a 30-h runtime.

The probability distribution $\tilde P$ is computed by uniformly distributing the estimated count rate (with or without accidental subtraction) from each leaf node across its constituent elements in $\tilde P$, followed by normalization.

## Supplementary information

Supplementary Information

## Data Availability

The data supporting the results presented in this manuscript is available from the corresponding author G.A.H. upon request.
