# Supplementary material for: Quantifying entanglement in a 68-billion-dimensional quantum state space
Source: Nat Commun. 2019 Jun 25;10:2785. doi: 10.1038/s41467-019-10810-z (PMC6592913; doi:10.1038/s41467-019-10810-z)
Supplement: Supplementary file 1 — Supplementary Information [file 41467_2019_10810_MOESM1_ESM.pdf]

Supplemental Material For Quantifying entanglement in a 68-billion dimensional quantum state space

James Schneeloch[1], Christopher C. Tison[1,2,3], Michael L. Fanto[1,4], Paul M. Alsing[1] & Gregory A. Howland[1,4,*]

[1] *Air Force Research Laboratory, Information Directorate, Rome, NY 13441 USA*

[2] *Department of Physics, Florida Atlantic University, Boca Raton, FL 33431 USA*

[3] *Quanterion Solutions Incorporated, Utica, NY 13502 USA*

[4] *Rochester Institute of Technology, Rochester, NY 14623 USA*

1 Proof that arbitrary coarse-graining cannot decrease conditional entropy

We are given two discrete probability distributions $P_1(X_\mathrm{A}, X_\mathrm{B})$ and $P_2(X_\mathrm{A}, X_\mathrm{B})$. We will also assume a permutation operation χ, that shuffles the outcomes of X_A and X_B. With this, we define the permuted distributions $P_1^{'}(X_\mathrm{A}, X_\mathrm{B})$ and $P_2^{'}(X_\mathrm{A}, X_\mathrm{B})$ as the result of permutation operator χ on $P_1(X_\mathrm{A}, X_\mathrm{B})$ and $P_2(X_\mathrm{A}, X_\mathrm{B})$, respectively.

The joint convexity of relative entropy states that given distributions $P_1, P_2, P_1^{'}$, and $P_2^{'}$, the following inequality holds:

$$\lambda\mathscr{D}(P_1||P_2) + (1-\lambda)\mathscr{D}(P_1^{'}||P_2^{'}) \geq$$
$$\geq \mathscr{D}(\lambda P_1 + (1-\lambda)P_1^{'}||\lambda P_2 + (1-\lambda)P_2^{'}) \tag{1}$$

where $\lambda \in [0, 1]$.

Next, we define the mixed probability distribution $\bar{P}_1 \equiv (\lambda P_1 + (1-\lambda)P_1^{'})$, and define $\bar{P}_2$ similarly. Since $P_1^{'}$ and $P_2^{'}$ are respectively related to P_1 and P_2 by the same permutation χ, we have that $\mathscr{D}(P_1||P_2) = \mathscr{D}(P_1^{'}||P_2^{'})$. Therefore, we obtain the inequality:

$$\mathscr{D}(P_1||P_2) \geq \mathscr{D}(\bar{P}_1||\bar{P}_2). \tag{2}$$

This result that mixing (i.e., majorization) cannot increase relative entropy has far reaching applications. In particular, coarse-graining is a form of majorization between adjacent elements in a probability distribution. Because all (Shannon) entropic functions can be expressed in terms of relative entropies, it immediately follows that:

$$H_{\bar{\mathrm{P}}}(X_{\mathrm{A}}) \geq H_{\mathrm{P}}(X_{\mathrm{A}}) \tag{3}$$

$$H_{\bar{\mathrm{P}}}(X_{\mathrm{A}}, X_{\mathrm{B}}) \geq H_{\mathrm{P}}(X_{\mathrm{A}}, X_{\mathrm{B}}) \tag{4}$$

$$H_{\bar{\mathrm{P}}}(X_{\mathrm{A}}|X_{\mathrm{B}}) \geq H_{\mathrm{P}}(X_{\mathrm{A}}|X_{\mathrm{B}}) \tag{5}$$

where the subscripts P and $\bar{\mathrm{P}}$ represent the probability distribution before and after coarse-graining, respectively. In addition, the mutual information and the conditional mutual information obey the inequalities

$$H_{\bar{\mathrm{P}}}(X_{\mathrm{A}} : X_{\mathrm{B}}) \leq H_{\mathrm{P}}(X_{\mathrm{A}} : X_{\mathrm{B}}) \tag{6}$$

$$H_{\bar{\mathrm{P}}}(X_{\mathrm{A}} : X_{\mathrm{B}}|X_{\mathrm{C}}) \leq H_{\mathrm{P}}(X_{\mathrm{A}} : X_{\mathrm{B}}|X_{\mathrm{C}}). \tag{7}$$

where again, the subscripts P and $\bar{\mathrm{P}}$ denote the true and coarse grained probability distribution, respectively. Furthermore, both the continuous mutual information $h(x_{\mathrm{A}} : x_{\mathrm{B}})$ and the continuous conditional mutual information $h(x_{\mathrm{A}} : x_{\mathrm{B}}|x_{\mathrm{C}})$ are expressible as high-resolution limits of corresponding discrete mutual informations. Because successive coarse grainings cannot increase these

quantities, the following inequalities hold between discrete and continuous mutual information

$$h(x_{\mathrm{A}} : x_{\mathrm{B}}) \geq H(X_{\mathrm{A}} : X_{\mathrm{B}}) \tag{8}$$

$$h(x_{\mathrm{A}} : x_{\mathrm{B}} | x_{\mathrm{C}}) \geq H(X_{\mathrm{A}} : X_{\mathrm{B}} | X_{\mathrm{C}}) \tag{9}$$

While the former inequality (8) can be found with alternative methods, the latter inequality (9) is new to the literature.

2 Proof of inequality 2

Inequality (2) derives from two fundamental properties of Shannon entropy. To expand notation, we have:

$$H(\mathbf{X}_{\mathrm{a}} | \mathbf{X}_{\mathrm{b}}) \equiv H(X_{\mathrm{a}}^{(1)}, ..., X_{\mathrm{a}}^{(d)} | X_{\mathrm{b}}^{(1)}, ..., X_{\mathrm{b}}^{(d)}) \tag{10}$$

First, is that the joint shannon entropy is less than or equal to the sum of the marginal entropies:

$$H(\mathbf{X}_{\mathrm{a}} | \mathbf{X}_{\mathrm{b}}) \leq \sum_{i=1}^{d} H(X_{\mathrm{a}}^{(i)} | X_{\mathrm{b}}^{(1)}, ..., X_{\mathrm{b}}^{(d)}) \tag{11}$$

Second, is that conditioning on additional variables cannot increase entropy, or conversely that removing conditioning variables cannot reduce entropy:

$$H(X_{\mathrm{a}}^{(i)} | X_{\mathrm{b}}^{(1)}, ..., X_{\mathrm{b}}^{(d)}) \leq H(X_{\mathrm{a}}^{(i)} | X_{\mathrm{b}}^{(i)}) \tag{12}$$

Together, this proves inequality (2):

$$H(\mathbf{X}_{\mathrm{a}} | \mathbf{X}_{\mathrm{b}}) \leq \sum_{i=1}^{d} H(X_{\mathrm{a}}^{(i)} | X_{\mathrm{b}}^{(i)}). \tag{13}$$

3 Monte Carlo error analysis

For the results shown in the manuscript, we used standard, first-order propagation-of-uncertainty for error analysis. Each coincidence-count measurement is assumed to have Poissonian uncertainty, and this uncertainty is analytically propagated through the analysis (e.g. $f(x_0 \pm \delta) = f(x_0) \pm \left(\frac{df}{dx}\right)_{x_0} \delta$).

To confirm the validity of our propagation-style error analysis, we also estimated our uncertainty with Monte Carlo simulations. This approach does not suffer any potential issues that may arise where our equations may not be sufficiently well-behaved for the first-order propagation of error. However, it does replace a simple analytical result with the need for computational simulations.

To perform Monte Carlo simulations, each coincidence count measurement is used to sample from a Poissonian distribution. Then, we follow our previously described process for generating joint-probability distributions (with or without accidental subtraction) and calculating the amount of entanglement. This process is repeated many times to see how the Poissonian counting statistics propagate to our final result.

In Supplemental Figure 1, we recreate Figure 3 from the main text using this approach with 100 trials. The error bars shown enclose two standard deviations. The uncertainties from this approach behave similarly to the analytic propagation-of-error used in the main manuscript, however the uncertainties are even smaller. The values obtained for the entanglement of formation are

$7.154 \pm .015$ $(7.112 \pm .0412)$ ebits with background subtraction and $3.459 \pm .012$ $(3.425 \pm .038)$ ebits, where the analytic result is given in parentheses. The two outcomes are in good agreement, with between two-times and four-times lower uncertainty with the Monte Carlo simulations.

4 Maximum possible entanglement that can be certified with this technique

For photon statistics contained within a finite window, the maximum possible entanglement our relation can characterize is when a pixel in the signal arm is correlated to only a single pixel in the idler arm, or when all conditional entropies are zero. In this case, the inequality reads:

$$E_{\mathrm{f}} \geq \log\left(\frac{(2\pi)^2}{\Delta x_{\mathrm{A}} \Delta y_{\mathrm{A}} \Delta k_{\mathrm{xA}} \Delta k_{\mathrm{yA}}}\right). \tag{14}$$

For perfect diagonal correlations, the number of measurements we need with our technique scales favorably with resolution, improving better with tighter correlations. For example, for $N \times N$ resolution in both position and momentum (assuming N is a power of two for simplicity), then one needs only about $12(N - \log_2(N) - 2)$ measurements, which, for $N = 512$ would be about 6096 measurements. This does not include the number of measurements needed to acquire this partitioning, which scales similarly. When the correlations are less tight, more pixels are required at maximum resolution, increasing this total.

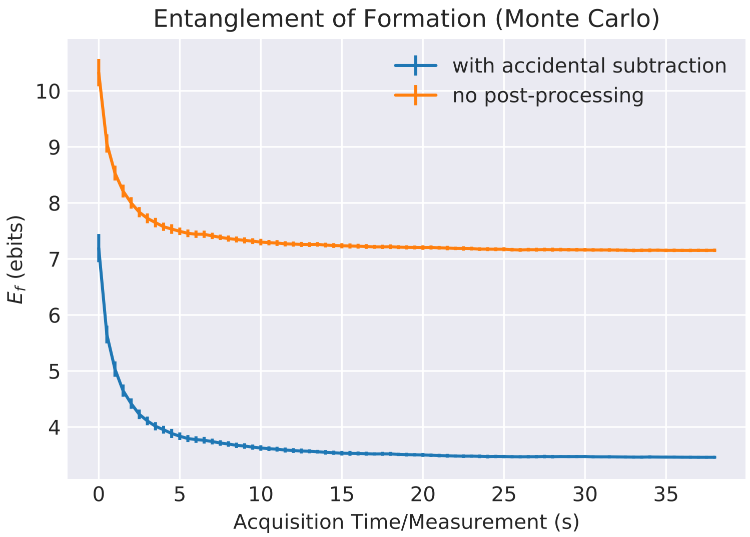

Supplemental Figure 1: **Supplemental: Entanglement quantification versus acquisition time with Monte Carlo uncertainty analysis** Measured coincidence counts are used to draw values from a Poisson distribution for 100 trials. Error bars enclose two standard deviations and are in good agreement with the analytical approach to error analysis used the main text (see Figure 3).
